# Response by Gonçalves et al to “Hemostatic Nets in Facelifts: A Systematic Review and Meta-Analysis of Postoperative Complications and Patient Outcomes” by Caimi et al

**DOI:** 10.1093/asjof/ojaf152

**Published:** 2025-12-19

**Authors:** Felipe Giraldo Alvarez Gonçalves, Leonardo Dexheimer da Silva, Emily Caroline da Fonseca Sampaio, Cristina Pires Camargo

Dear Editor,

We read with interest the article *Hemostatic Nets in Facelifts: A Systematic Review and Meta-Analysis of Postoperative Complications and Patient Outcomes*, by Caimi et al.^1^ We commend the authors for addressing a relevant clinical question. However, some methodological limitations deserve consideration.

Among the 5 meta-analyzed studies, two^2,3^ originated from the same author and reported overlapping populations, yet, were treated as independent. Treating overlapping cohorts as independent violates the assumption of independence underpinning meta-analysis and likely results in artificially narrow confidence intervals. Nevertheless, heterogeneity for hematoma was substantial and would almost certainly be higher if duplicates were properly addressed.

Although the authors used a random-effects model, they did not investigate sources of heterogeneity. No meta-regressions, leave-one-out analyses, or subgroup analyses were performed to explore between-study differences.

Additionally, although the title states that the review evaluates hemostatic nets, several included studies did not employ the technique as originally described. Auersvald's^2,3^ hemostatic net consists of a full grid of continuous transcutaneous quilting sutures, whereas three included studies (50% if the 2 Auersvald cohorts^2,3^ are counted as one) used only internal quilting sutures or a combination of internal and interrupted external stitches, not continuous grids. Pooling these heterogeneous procedures under the label “hemostatic net” misrepresents the actual interventions assessed.

Also, O’Daniel et al,^4^ which evaluated hemostatic nets in facelift surgery, reported postoperative complications, and is even cited in the discussion, was excluded. This study clearly meets the stated inclusion criteria. Omitting it while retaining overlapping cohorts introduces selection bias and undermines the claim of a comprehensive evidence base.

In conclusion, we believe that the study is constrained by (1) high and unexplained heterogeneity, (2) an imprecise definition of the intervention, (3) selective inclusion, and (4) reliance on overlapping cohorts in the meta-analysis.
